# An adaptive discretized RNN algorithm for posture collaboration motion control of constrained dual-arm robots

**DOI:** 10.3389/fnbot.2024.1406604

**Published:** 2024-05-22

**Authors:** Yichen Zhang, Yu Han, Binbin Qiu

**Affiliations:** School of Intelligent Systems Engineering, Sun Yat-sen University, Shenzhen, China

**Keywords:** dual-arm robot, dual-arm posture collaboration motion control (DAPCMC), time-variant equation system (TVES), adaptive Taylor-type discretized recurrent neural network (ATT-DRNN), joint-limit conversion strategy

## Abstract

Although there are many studies on repetitive motion control of robots, few schemes and algorithms involve posture collaboration motion control of constrained dual-arm robots in three-dimensional scenes, which can meet more complex work requirements. Therefore, this study establishes the minimum displacement repetitive motion control scheme for the left and right robotic arms separately. On the basis of this, the design mentality of the proposed dual-arm posture collaboration motion control (DAPCMC) scheme, which is combined with a new joint-limit conversion strategy, is described, and the scheme is transformed into a time-variant equation system (TVES) problem form subsequently. To address the TVES problem, a novel adaptive Taylor-type discretized recurrent neural network (ATT-DRNN) algorithm is devised, which fundamentally solves the problem of calculation accuracy which cannot be balanced well with the fast convergence speed. Then, stringent theoretical analysis confirms the dependability of the ATT-DRNN algorithm in terms of calculation precision and convergence rate. Finally, the effectiveness of the DAPCMC scheme and the excellent convergence competence of the ATT-DRNN algorithm is verified by a numerical simulation analysis and two control cases of dual-arm robots.

## 1 Introduction

With the continuous development of electronic information technology, robots, as a key carrier in the realm of artificial intelligence, have been assuming a progressively substantial role in manufacturing (Arents and Greitans, 2022), healthcare (Khan et al., 2020), service industries (McCartney and McCartney, 2020), and beyond (Cheng et al., 2023; Tanyıldızı, 2023; Yang et al., 2023; Liufu et al., 2024), bringing numerous conveniences to human life and work. Many scholars are focusing their attention on robotics research field.

A robotic arm is a mechanical device composed of multiple linked joints, typically equipped with various end-effectors based on the requirements of the work environment. By calculating and adjusting the rotational changes of each joint, the end-effector can be controlled to perform various movements in a predetermined manner, such as position and orientation, thereby accomplishing tasks. For instance, the MATLAB program and particle swarm optimization were utilized for the trajectory planning of the robotic arm (Ekrem and Aksoy, 2023); Chico et al. (2021) employed a hand gesture recognition system and the inertial measurement unit to control the position and orientation of a virtual robotic arm. A target admittance model was designed in the joint space for hands-on procedures that can be applied in all commercially available general-purpose robotic arms with six or more DOF (Kastritsi and Doulgeri, 2021).

Due to the escalating complexity of task environments, single robotic arms frequently encounter challenges in effectively completing tasks, which highlights the advantages of dual robotic arms in collaborative and efficient task execution. For example, Jiang et al. (2022) presented an adaptive control method for a dual-arm robot to perform bimanual tasks under modeling uncertainties. Bombile and Billard (2022) designed a unified motion generation algorithm that enables a dual-arm robot to grab and release objects quickly. Wang et al. (2023) proposed a sliding mode controller with good robustness against the model uncertainties to capture and stabilize a spinning target in 3D space by a dual-arm space robot.

However, some of the methods mentioned above do not take into account the actual physical constraints of the robotic arms during initial modeling (e.g., Bombile and Billard, 2022; Jiang et al., 2022). This greatly limits the application scenarios of these algorithms and is inconsistent with the real working conditions of the robotic arms. Furthermore, the physical limitations of robotic arms typically pertain to constraints on joint angle and velocity. These constraints do not reside at the same constraint level, thus there are substantial computational challenges when attempting to address them collectively. An optimal approach entails a series of conversion strategies to harmonize these distinct hierarchical constraints to a congruous level (Zhang and Zhang, 2013) (e.g., velocity level). By implementing this approach, the constraints can be effectively unified and dealt without compromising their intended meaning. Some scholars (e.g., Li, 2020) have crafted novel approaches to these conversion strategies stemming from this foundation. Nevertheless, in the process, they have introduced too many supplementary parameters, rendering the strategies less straightforward for apprehension. Additionally, certain studies focus on the control of dual robotic arms based on 2D space, considerably limiting the operating range of robotic arms (Stolfi et al., 2017; Yang S. et al., 2020; Yang et al., 2021).

In recent years, with the rapid advancement of neural network research, many scholars have been committed to applying its formidable nonlinear modeling capability and efficient parallel computing ability to the domain of robotic arm motion control (Wang et al., 2021; Jin et al., 2024). This endeavor has given rise to a special kind of neural network known as the RNN (Xiao et al., 2021; Yan et al., 2022; Fu et al., 2023). For example, Xiao et al. (2021) proposed a noise-enduring and finite-time convergent design formula is suggested to establish a novel RNN. Fu et al. (2023) presented a gradient-feedback RNN to solve the unconstrained time-variant convex optimization problem.

To facilitate the calculation on computers and other digital hardware devices, some scholars focus on discretizing conventional CRNN models through time discretization techniques, leading to the development of DRNN algorithms (Liao et al., 2016; Liu et al., 2023a,b; Shi et al., 2023). The technique of second-order Taylor expansion was used to deal with the discrete time-variant nonlinear system, and a DRNN algorithm was proposed subsequently (Shi et al., 2023). Liao et al. (2016) proposed two Taylor-type DRNN algorithms on account of the Taylor-type formula to perform online dynamic equality-constrained quadratic programming. Liu et al. (2023a) designed a Taylor-type DRNN algorithm based on Taylor-type discrete scheme with smaller TE. It is worth noting that higher accuracy requirements often make the discretization formulas more complicated, inevitably leading to a large amount of computation and increasing the cost of actual production applications. After overall consideration, this study proposes an adaptive DRNN algorithm based on a three-step general Taylor-type discretization formula with an adaptive sampling period introduced, which is of high enough precision for practical applications.

Typically, due to the use of fixed sampling periods and fixed convergence factors in the conventional DRNN algorithms mentioned above, it is difficult for them to achieve a balance in computational precision and convergence rate, resulting in limited algorithmic dynamic and convergence performance. Therefore, some researchers have tried to introduce various adaptive mechanisms into model/algorithm design (Song et al., 2008; Yang M. et al., 2020; Dai et al., 2022; Cai and Yi, 2023). For example, Yang M. et al. (2020) proposed two discretized RNN algorithms with an adaptive Jacobian matrix. Cai and Yi (2023) developed an adaptive gradient-descent-based RNN model to solve time-variant problems based on the Lyapunov theory. Dai et al. (2022) proposed a hybrid RNN model by introducing a fuzzy adaptive control strategy to generate a fuzzy adaptive factor that can change its size adaptively according to the RE. Song et al. (2008) proposed a robust adaptive gradient-descent training algorithm based on an RNN hybrid training concept in discrete-time domain.

In light of the aforementioned circumstances, this study formulates a DAPCMC scheme in 3D space based on the dual-arm robot system and the new JLCS. Subsequently, a novel ATT-DRNN algorithm with adaptive sampling period and adaptive convergence factor is devised to effectively face the challenge of achieving a dynamic balance between great computational precision and rapid convergence rate. When compared with the CTT-DRNN algorithm and the CET-DRNN algorithm, the proposed ATT-DRNN algorithm demonstrates outstanding computational precision and rapid convergence rate. To demonstrate the features and strengths of the proposed ATT-DRNN algorithm, Table 1 shows the comparisons among distinct methods for the motion control of robots.

**Table 1:** Comparisons among distinct methods for motion control of robots.

**Method**	**Posture control**	**Inequality constraint**	**Discretized handling**	**Adaptive mechanism**	**Applicable scene**	**Robotic arm number**
Jiang et al. (2022)	No	No	No	Yes	2D	Dual
Yang et al. (2021)	Yes	Yes	Yes	No	2D	Dual
Yang S. et al. (2020)	No	Yes	No	No	2D	Dual
Fu et al. (2023)	No	No	No	No	3D	Single
Shi et al. (2023)	No	No	Yes	No	2D	Single
Liao et al. (2016)	No	No	Yes	No	2D	Single
Wu and Zhang (2023)	Yes	No	Yes	Yes	3D	Single
Yang M. et al. (2020)	No	No	Yes	Yes	3D	Single
ATT-DRNN	Yes	Yes	Yes	Yes	3D	Dual

The remainder of this study consists of four parts. Section 2 formulates the DAPCMC scheme and designs the ATT-DRNN algorithm. Section 3 presents the theoretical analyses of the proposed ATT-DRNN algorithm. Section 4 provides illustrative examples, and Section 5 concludes this study. Finally, the primary contributions/novelties of this paper can be summarized as follows.

1) Distinguishing from common dual-arm robot motion control schemes in 2D space, a novel construction methodology of the DAPCMC scheme in 3D space is provided, which can make a spatial dual-arm robot collaboratively execute repetitive tracking of a desired trajectory while adhering to a predetermined posture.
2) Distinguishing from existing strategies, an innovative JLCS is proposed, which has a ubiquitously differentiable and more succinct expression.
3) Distinguishing from conventional discretization methods, an innovative ATT-DRNN algorithm is engineered to address the DAPCMC scheme, which introduces a new adaptive convergence factor and sampling period to guarantee a notable convergence rate and exceptional convergence precision.
4) Distinguishing from the simple path-tracking task of single-arm robots, the posture collaboration motion control experiments of a UR5 dual-arm robot with the joint-angle and joint-velocity bound constraints considered substantiate the effectiveness of the proposed DAPCMC scheme and the outstanding convergence capability of the proposed ATT-DRNN algorithm.

## 2 Scheme formulation and algorithm design

This section describes how to construct a DAPCMC scheme that can be converted into a TVES problem and processed by the proposed ATT-DRNN algorithm.

### 2.1 Rudimentary knowledge

For the convenience of comprehension, let us construct a single robot arm motion control scheme with *n* DOF, which takes into account joint physical limits and can simultaneously ensure position control and orientation control during the MDRMC. Specifically, such a scheme can be described as below:


(1) $$\begin{array}{lll} {\text{min}.}_{\dot{z}\left(t\right)} & \; & \frac{1}{2}{\dot{z}}^{\text{T}}\left(t\right)U\left(t\right)\dot{z}\left(t\right)+\varphi^{\text{T}}\left(t\right)\dot{z}\left(t\right), \end{array}$$

(2) $$\begin{array}{lll} \text{s.t.} & \; & J_{1}\left(z\left(t\right)\right)\dot{z}\left(t\right)={\dot{\Upsilon }}_{\text{I}}\left(t\right)-\alpha \left[\Upsilon_{\text{R}}\left(t\right)-\Upsilon_{\text{I}}\left(t\right)\right], \end{array}$$

(3) $$\begin{array}{ll} \; & J_{2}\left(z\left(t\right)\right)\dot{z}\left(t\right)={\dot{o}}_{\text{I}}\left(t\right)-\beta \left[o_{\text{R}}\left(t\right)-o_{\text{I}}\left(t\right)\right], \end{array}$$

(4) $$\begin{array}{ll} \; & z^{-}\leq z\left(t\right)\leq z^{+}, \end{array}$$

(5) $$\begin{array}{ll} \; & {\dot{z}}^{-}\leq \dot{z}\left(t\right)\leq {\dot{z}}^{+}, \end{array}$$

where superscript ^T^ represents the transpose operator; $z\left(t\right)={\left[{\dot{z}}_{1}\left(t\right),{\dot{z}}_{2}\left(t\right),...,{\dot{z}}_{n}\left(t\right)\right]}^{\text{T}}\in {\mathbb{R}}^{n}$ indicates the angle values of the robotic joints, and $\dot{z}\left(t\right)\in {\mathbb{R}}^{n}$ means the angular velocities of the robotic joints; matrix $U\left(t\right)=I_{n\times n}\in {\mathbb{R}}^{n\times n}$ is an identity matrix; vector $\varphi \left(t\right)=\xi \left[z\left(t\right)-z\left(0\right)\right]\in {\mathbb{R}}^{n}$ with design parameter ξ > 0 and z(0) means the initial joint-angle vector; $J_{1}\left(z\left(t\right)\right)\in {\mathbb{R}}^{3\times n}$ and $J_{2}\left(z\left(t\right)\right)\in {\mathbb{R}}^{3\times n}$ represent the position Jacobian matrix and the orientation Jacobian matrix, respectively; $\Upsilon_{\text{I}}\left(t\right)\in {\mathbb{R}}^{3}$ and $\Upsilon_{\text{R}}\left(t\right)\in {\mathbb{R}}^{3}$ represent the ideal path and the real position of the end-executor, separately; $o_{\text{I}}\left(t\right)\in {\mathbb{R}}^{3}$ and $o_{\text{R}}\left(t\right)\in {\mathbb{R}}^{3}$ represent the ideal orientation and the real orientation of the end-executor, respectively; α > 0 and β > 0 are both the error-feedback gains; $z^{\pm }$ and ${\dot{z}}^{\pm }$ denote the upper and lower limits of z(t) and $\dot{z}\left(t\right)$, separately.

*Remark 2.1:* In accordance with previous experience (Zhang and Zhang, 2013), when *t* → ∞, the objective function (1) at the joint-velocity level is equivalent to ${\left||z\left(t\right)-z\left(0\right)|\right|}_{2}^{2}/2$ at the joint-angle level, where the design parameter ξ > 0 ought to be adjusted as large as allowed by the manipulator conditions. Note that the robot arm's repetitive motion planning scheme under minimal displacement can be regarded as an optimization objective that can be resolved at the joint-velocity level.

*Remark 2.2:* Referring to the contributions of previous scholars (Yang et al., 2021), the equality constraint (2) at the joint-velocity level is equivalent to $f\left(z\left(t\right)\right)=\Upsilon_{\text{I}}\left(t\right)$ at the joint-angle level, when *t* → ∞ and the error-feedback gain α > 0 is at an appropriate value, where ***f***(·):ℝ^*n*^ → ℝ^3^ represents the forward kinematics mapping function of a robotic arm.

*Remark 2.3:* Similarly, the equality constraint (3) at the joint-velocity level is equivalent to $g\left(z\left(t\right)\right)=o_{\text{I}}\left(t\right)$ at the joint-angle level, when *t* → ∞ and the error-feedback gain β > 0 is at an appropriate value, where nonlinear function $g\left(z\left(t\right)\right)=o_{\text{R}}\left(t\right)={\left[o_{\text{Rx}}\left(t\right),o_{\text{Ry}}\left(t\right),o_{\text{Rz}}\left(t\right)\right]}^{\text{T}}\in {\mathbb{R}}^{3}$ and the 2-norm of the real orientation vector ***o***_R_(*t*) satisfies ||***o***_R_(*t*)||_2_ = 1.

Note that the inequality constraint (4) is at the joint-angle level of the system. In order to integrate inequality constraints (4) and (5) of distinct constraint levels into a unified formulation at the joint-velocity level as below:


(6) $$\begin{array}{l} {℧}^{-}\left(t\right)\leq \dot{z}\left(t\right)\leq {℧}^{+}\left(t\right), \end{array}$$

previous studies (Zhang and Zhang, 2013; Zhang et al., 2018; Li, 2020; Li et al., 2023; Qiu et al., 2023) supply a large number of JLCSs.

Nevertheless, the JLCS in Zhang and Zhang (2013) is unable to guarantee **℧**^−^(*t*) or **℧**^+^(*t*) to be differentiable anywhere. Meanwhile, as regard to the JLCS in Li (2020), **℧**^−^(*t*) and **℧**^+^(*t*) are designed as piecewise functions, respectively, and complex compound functions are embedded in them. In addition, the JLCS in Li et al. (2023); Qiu et al. (2023) adopt numerous design parameters and construct pretty complex expressions.

Therefore, as one of the contributions of this study, we provide a new JLCS. The *i*th (*i* = 1, 2, ..., *n*) elements of **℧**^−^(*t*) and **℧**^+^(*t*) in (6) are designed as follows:


$$\begin{array}{l} \{\begin{array}{cc} {℧}_{i}^{-}\left(t\right)={\dot{z}}_{i}^{-}\exp\left[\frac{\gamma {\dot{z}}_{i}\left(t\right)}{{\dot{z}}_{i}\left(t\right)-{\dot{z}}_{i}^{-}+\varepsilon_{1}}\right],\varepsilon_{1}\to 0_{+}, & \text{(7)}\\ {℧}_{i}^{+}\left(t\right)={\dot{z}}_{i}^{+}\exp\left[\frac{\gamma {\dot{z}}_{i}\left(t\right)}{{\dot{z}}_{i}\left(t\right)-{\dot{z}}_{i}^{+}+\varepsilon_{2}}\right],\varepsilon_{2}\to 0_{-}, & \text{(8)} \end{array} \end{array}$$

where ${\dot{z}}_{i}\left(t\right),{\dot{z}}_{i}^{-},{\dot{z}}_{i}^{+},{\dot{z}}_{i}^{-},{\dot{z}}_{i}^{+}$ denote the *i*th element of $z\left(t\right),z^{-},z^{+},{\dot{z}}^{-},{\dot{z}}^{+}$ in (4) and (5), separately; ε_1_ and ε_2_ are both non-zero minimum terms to ensure that the above equations are able to differentiable everywhere; design parameter γ∈(0, 1) should be as small as possible.

**Figure 1:**
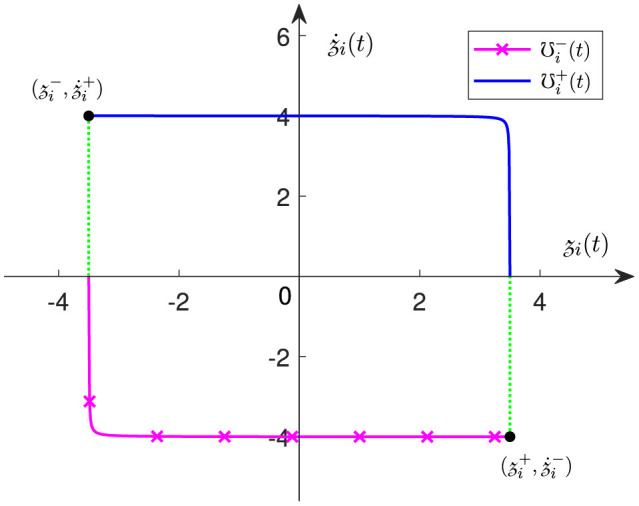
The relationship between the *i*th joint angle ${\dot{z}}_{i}\left(t\right)$ and the *i*th joint velocity ${\dot{z}}_{i}\left(t\right)$ in the proposed JLCS (7)–(8), with *i* = 1, 2, ..., *n*.

*Remark 2.4:* To present the proposed JLCS (7)-(8) more specifically, Figure 1 exhibits the relationship between the *i*th joint angle ${\dot{z}}_{i}\left(t\right)$ and the *i*th joint velocity ${\dot{z}}_{i}\left(t\right)$. It is worth noting that, when the joint approaches its lower or upper limit, the value of γ has a crucial effect on the changing rate of the joint-velocity boundary.

### 2.2 DAPCMC scheme

Finally, upon the previous section, we construct a dual-arm collaborative control system consisting of the LA and RA.

#### 2.2.1 LA collaborative control subsystem

According to (1)–(5), we construct the MDRMC scheme for the *n*-DOF LA as follows:


(9) $$\begin{array}{l} {\text{min}.}_{{\dot{z}}_{L}\left(t\right)}\frac{1}{2}{\dot{z}}_{L}^{\text{T}}\left(t\right)U_{L}\left(t\right){\dot{z}}_{L}\left(t\right)+\varphi_{L}^{\text{T}}\left(t\right){\dot{z}}_{L}\left(t\right), \end{array}$$

(10) $$\begin{array}{l} \text{s.t.}J_{1L}\left(z_{L}\left(t\right)\right){\dot{z}}_{L}\left(t\right)={\dot{\Upsilon }}_{\text{I}L}\left(t\right)\\ -\alpha_{L}\left[\Upsilon_{\text{R}L}\left(t\right)-\Upsilon_{\text{I}L}\left(t\right)\right], \end{array}$$

(11) $$\begin{array}{l} J_{2L}\left(z_{L}\left(t\right)\right){\dot{z}}_{L}\left(t\right)={\dot{o}}_{\text{I}L}\left(t\right)\\ -\beta_{L}\left[o_{\text{R}L}\left(t\right)-o_{\text{I}L}\left(t\right)\right], \end{array}$$

(12) $$\begin{array}{l} z_{L}^{-}\leq z_{L}\left(t\right)\leq z_{L}^{+}, \end{array}$$

(13) $$\begin{array}{ll} {\dot{z}}_{L}^{-}\leq {\dot{z}}_{L}\left(t\right)\leq {\dot{z}}_{L}^{+}, & \; \end{array}$$

where the subscript _ℒ_ denotes the LA; vector $z_{L}\left(t\right)={\left[{\dot{z}}_{L1}\left(t\right),{\dot{z}}_{L2}\left(t\right),...,{\dot{z}}_{Ln}\left(t\right)\right]}^{\text{T}}\in {\mathbb{R}}^{n}$; vector $\varphi_{L}\left(t\right)=\xi_{L}\left[z_{L}\left(t\right)-z_{L}\left(0\right)\right]\in {\mathbb{R}}^{n}$ with the design parameter $\xi_{L}>0$ and $z_{L}\left(0\right)$ means the LA's initial joint-angle vector. Additionally, the meanings represented by the other symbols are similar to those in the MDRMC scheme (1)–(5).

Moreover, according to the JLCS (7)–(8), (12) and (13) in the LA's MDRMC scheme can be converted into the following form:


(14) $$\begin{array}{l} {℧}_{L}^{-}\left(t\right)\leq {℧}_{L}\left(t\right)\leq {℧}_{L}^{+}\left(t\right), \end{array}$$

where ${℧}_{L}\left(t\right)={\dot{z}}_{L}\left(t\right)={\left[{\dot{z}}_{L1}\left(t\right),{\dot{z}}_{L2}\left(t\right),,...,{\dot{z}}_{Ln}\left(t\right)\right]}^{\text{T}}\in {\mathbb{R}}^{n}$; the upper and lower limit values of ${℧}_{L}^{-}\left(t\right)$ and ${℧}_{L}^{+}\left(t\right)$ are correspondence to those of **℧**^−^(*t*) and **℧**^+^(*t*) in the JLCS (7)-(8).

Furthermore, by reorganizing the LA's MDRMC scheme (9)-(13), we obtain the LA collaborative control subsystem scheme, which has a briefer representation:


(15) $$\begin{array}{lll} {\text{min}.}_{{℧}_{L}\left(t\right)} & \; & \frac{1}{2}{℧}_{L}^{\text{T}}\left(t\right)U_{L}\left(t\right){℧}_{L}\left(t\right)+\varphi_{L}^{\text{T}}\left(t\right){℧}_{L}\left(t\right), \end{array}$$

(16) $$\begin{array}{lll} \text{s.t.} & \; & A_{L}\left(t\right){℧}_{L}\left(t\right)=c_{L}\left(t\right), \end{array}$$

(17) $$\begin{array}{ll} \; & B_{L}\left(t\right){℧}_{L}\left(t\right)\leq d_{L}\left(t\right), \end{array}$$

where matrices $A_{L}\left(t\right)\in {\mathbb{R}}^{6\times n}$ and $B_{L}\left(t\right)\in {\mathbb{R}}^{2n\times n}$ and vectors $c_{L}\left(t\right)\in {\mathbb{R}}^{6}$ and $d_{L}\left(t\right)\in {\mathbb{R}}^{2n}$ are expressed as below:


$$\begin{array}{l} A_{L}\left(t\right)=\left[\begin{array}{c} J_{1L}\left(z_{L}\left(t\right)\right)\\ J_{2L}\left(z_{L}\left(t\right)\right)\\ \; \end{array}\right],\\ c_{L}\left(t\right)=\left[\begin{array}{c} {\dot{\Upsilon }}_{\text{I}L}\left(t\right)-\alpha_{L}\left[\Upsilon_{\text{R}L}\left(t\right)-\Upsilon_{\text{I}L}\left(t\right)\right]\\ {\dot{o}}_{\text{I}L}\left(t\right)-\beta_{L}\left[o_{\text{R}L}\left(t\right)-o_{\text{I}L}\left(t\right)\right] \end{array}\right]\\ B_{L}\left(t\right)=\left[\begin{array}{c} I_{n\times n}\\ -I_{n\times n}\\ \; \end{array}\right],\;d_{L}\left(t\right)=\left[\begin{array}{c} {℧}_{L}^{+}\left(t\right)\\ -{℧}_{L}^{-}\left(t\right)\\ \; \end{array}\right]. \end{array}$$

#### 2.2.2 RA collaborative control subsystem

Similar to the (2.2.1), the MDRMC scheme for the *n*-DOF RA is follows:


(18) $$\begin{array}{l} {\text{min}.}_{{\dot{z}}_{R}\left(t\right)}\frac{1}{2}{\dot{z}}_{R}^{\text{T}}\left(t\right)U_{R}\left(t\right){\dot{z}}_{R}\left(t\right)+\varphi_{R}^{\text{T}}\left(t\right){\dot{z}}_{R}\left(t\right), \end{array}$$

(19) $$\begin{array}{l} \text{s.t.}J_{1R}\left(z_{R}\left(t\right)\right){\dot{z}}_{R}\left(t\right)={\dot{\Upsilon }}_{\text{I}R}\left(t\right)\\ -\alpha_{R}\left[\Upsilon_{\text{R}R}\left(t\right)-\Upsilon_{\text{I}R}\left(t\right)\right], \end{array}$$

(20) $$\begin{array}{l} J_{2R}\left(z_{R}\left(t\right)\right){\dot{z}}_{R}\left(t\right)={\dot{o}}_{\text{I}R}\left(t\right)\\ -\beta_{R}\left[o_{\text{R}R}\left(t\right)-o_{\text{I}R}\left(t\right)\right], \end{array}$$

(21) $$\begin{array}{ll} z_{R}^{-}\leq z_{R}\left(t\right)\leq z_{R}^{+}, & \; \end{array}$$

(22) $$\begin{array}{ll} {\dot{z}}_{R}^{-}\leq {\dot{z}}_{R}\left(t\right)\leq {\dot{z}}_{R}^{+}, & \; \end{array}$$

where the subscript _ℛ_ denotes the RA; vector $z_{R}\left(t\right)={\left[{\dot{z}}_{R1}\left(t\right),{\dot{z}}_{R2}\left(t\right),...,{\dot{z}}_{Rn}\left(t\right)\right]}^{\text{T}}\in {\mathbb{R}}^{n}$; vector $\varphi_{R}\left(t\right)=\xi_{R}\left[z_{R}\left(t\right)-z_{R}\left(0\right)\right]\in {\mathbb{R}}^{n}$ with design parameter $\xi_{R}>0$ and $z_{R}\left(0\right)$ means the RA's initial joint-angle vector. Additionally, the meanings represented by the other symbols are similar to those in the MDRMC scheme for the LA (9)–(13).

Similarly, (21) and (22) in the RA's MDRMC scheme can be transformed into the following form:


(23) $$\begin{array}{l} {℧}_{R}^{-}\left(t\right)\leq {℧}_{R}\left(t\right)\leq {℧}_{R}^{+}\left(t\right), \end{array}$$

where ${℧}_{R}\left(t\right)={\dot{z}}_{R}\left(t\right)={\left[{\dot{z}}_{R1}\left(t\right),{\dot{z}}_{R2}\left(t\right),,...,{\dot{z}}_{Rn}\left(t\right)\right]}^{\text{T}}\in {\mathbb{R}}^{n}$; the upper and lower limit values of ${℧}_{R}^{-}\left(t\right)$ and ${℧}_{R}^{+}\left(t\right)$ are parallelism to those of ${℧}_{L}^{-}\left(t\right)$ and ${℧}_{L}^{+}\left(t\right)$ in the LA's JLCS (14).

Then, by reorganizing the RA's MDRMC scheme (18)–(22), we obtain the RA collaborative control subsystem scheme:


(24) $$\begin{array}{lll} {\text{min}.}_{{℧}_{R}\left(t\right)} & \; & \frac{1}{2}{℧}_{R}^{\text{T}}\left(t\right)U_{R}\left(t\right){℧}_{R}\left(t\right)+\varphi_{R}^{\text{T}}\left(t\right){℧}_{R}\left(t\right), \end{array}$$

(25) $$\begin{array}{lll} \text{s.t.} & \; & A_{R}\left(t\right){℧}_{R}\left(t\right)=c_{R}\left(t\right), \end{array}$$

(26) $$\begin{array}{ll} \; & B_{R}\left(t\right){℧}_{R}\left(t\right)\leq d_{R}\left(t\right), \end{array}$$

where matrices $A_{R}\left(t\right)\in {\mathbb{R}}^{6\times n}$ and $B_{R}\left(t\right)\in {\mathbb{R}}^{2n\times n}$ and vectors $c_{R}\left(t\right)\in {\mathbb{R}}^{6}$ and $d_{R}\left(t\right)\in {\mathbb{R}}^{2n}$ are expressed as follows:


$$\begin{array}{l} A_{R}\left(t\right)=\left[\begin{array}{c} J_{1R}\left(z_{R}\left(t\right)\right)\\ J_{2R}\left(z_{R}\left(t\right)\right)\\ \; \end{array}\right],\\ c_{R}\left(t\right)=\left[\begin{array}{c} {\dot{\Upsilon }}_{\text{I}R}\left(t\right)-\alpha_{R}\left[\Upsilon_{\text{R}R}\left(t\right)-\Upsilon_{\text{I}R}\left(t\right)\right]\\ {\dot{o}}_{\text{I}R}\left(t\right)-\beta_{R}\left[o_{\text{R}R}\left(t\right)-o_{\text{I}R}\left(t\right)\right] \end{array}\right]\\ B_{R}\left(t\right)=\left[\begin{array}{c} I_{n\times n}\\ -I_{n\times n}\\ \; \end{array}\right],\;d_{R}\left(t\right)=\left[\begin{array}{c} {℧}_{R}^{+}\left(t\right)\\ -{℧}_{R}^{-}\left(t\right)\\ \; \end{array}\right]. \end{array}$$

Furthermore we combine the LA collaborative control subsystem scheme (15)–(17) with the RA collaborative control subsystem scheme (24)–(26) to obtain a complete DAPCMC scheme, which is also a TVQP problem:


(27) $$\begin{array}{lll} {\text{min}.}_{℧\left(t\right)} & \; & \frac{1}{2}{℧}^{\text{T}}\left(t\right)U\left(t\right)℧\left(t\right)+\varphi^{\text{T}}\left(t\right)℧\left(t\right), \end{array}$$

(28) $$\begin{array}{lll} \text{s.t.} & \; & A\left(t\right)℧\left(t\right)=c\left(t\right), \end{array}$$

(29) $$\begin{array}{ll} \; & B\left(t\right)℧\left(t\right)\leq d\left(t\right), \end{array}$$

where


$$\begin{array}{l} A\left(t\right)=\left[\begin{array}{cc} A_{L}\left(t\right) & 0\\ 0 & A_{R}\left(t\right)\\ \; \end{array}\right]\in {\mathbb{R}}^{12\times 2n},\;℧\left(t\right)=\left[\begin{array}{c} {℧}_{L}\left(t\right)\\ {℧}_{R}\left(t\right)\\ \; \end{array}\right]\in {\mathbb{R}}^{2n},\\ c\left(t\right)=\left[\begin{array}{c} c_{L}\left(t\right)\\ c_{R}\left(t\right)\\ \; \end{array}\right]\in {\mathbb{R}}^{12}\\ B\left(t\right)=\left[\begin{array}{cc} B_{L}\left(t\right) & 0\\ 0 & B_{R}\left(t\right)\\ \; \end{array}\right]\in {\mathbb{R}}^{4n\times 2n},\;d\left(t\right)=\left[\begin{array}{c} d_{L}\left(t\right)\\ d_{R}\left(t\right)\\ \; \end{array}\right]\in {\mathbb{R}}^{4n},\\ \;\varphi \left(t\right)=\left[\begin{array}{c} \varphi_{L}\left(t\right)\\ \varphi_{R}\left(t\right)\\ \; \end{array}\right]\in {\mathbb{R}}^{2n}\\ U\left(t\right)=\left[\begin{array}{cc} U_{L}\left(t\right) & 0\\ 0 & U_{R}\left(t\right)\\ \; \end{array}\right]\in {\mathbb{R}}^{2n\times 2n}. \end{array}$$

In order to resolve the proposed DAPCMC scheme (27)–(29), that is, to seek the optimal solution to the TVQP problem (27)–(29), it is necessary for us to concentrate on how to translate such a TVQP problem (27)–(29) into a more computationally convenient TVES problem. After that, solving the TVES problem is tantamount to finding the optimal solution to the TVQP problem (27)–(29).

With reference to Wei et al. (2022), the optimal solution to the TVQP problem (27)–(29) can be obtained by dealing with the following TVES problem:


(30) $$\begin{array}{l} H\left(t\right)\chi \left(t\right)+g\left(t\right)=0, \end{array}$$

where the coefficient matrix $H\left(t\right)\in {\mathbb{R}}^{\varpi \times \varpi }$ and the vectors **χ**(*t*)∈ℝ^ϖ^ and $g\left(t\right)\in {\mathbb{R}}^{\varpi }$ can be described as follows:


$$\begin{array}{l} H\left(t\right)=\left[\begin{array}{ccc} U\left(t\right) & A^{\text{T}}\left(t\right) & B^{\text{T}}\left(t\right)\\ A\left(t\right) & 0_{12\times 12} & 0_{12\times 4n}\\ -B\left(t\right) & 0_{4n\times 12} & I_{4n\times 4n} \end{array}\right]\\ \chi \left(t\right)=\left[\begin{array}{c} ℧\left(t\right)\\ \lambda \left(t\right)\\ \mu \left(t\right) \end{array}\right],\;g\left(t\right)=\left[\begin{array}{c} \varphi \left(t\right)\\ -c\left(t\right)\\ r\left(t\right) \end{array}\right] \end{array}$$

where the Lagrange multiplier **λ**(*t*)∈ℝ^12^ is connected with the equality constraint (28) and the Lagrange multiplier **μ**(*t*)∈ℝ^4*n*^ connected with the inequality constraint (29); $r\left(t\right)=d\left(t\right)-\sqrt{v\left(t\right)\circ v\left(t\right)+\mu \left(t\right)\circ \mu \left(t\right)+\varepsilon_{3}}$ and ***v***(*t*) = ***d***(*t*)−***B***(*t*)**℧**(*t*); ∘ bespeaks the Hadamard product operator; **ε**_3_ → 0_+_ and ϖ = 6*n* + 12.

In other words, as long as we can explore the solution **χ**(*t*) suitable for the TVES problem (30), it means that we have found the optimal solution to the TVQP problem (27)–(29); Next, we will explain the derivation of the ATT-DRNN algorithm and employ it to work out the TVES problem (30), the TVQP problem (27)–(29), and the proposed DAPCMC scheme (27)–(29).

### 2.3 Algorithm design

First, we set up the following vector-valued EE in the light of the TVES problem (30):


(31) $$\begin{array}{l} e\left(t\right)=H\left(t\right)\chi \left(t\right)+g\left(t\right). \end{array}$$

Finally, we utilize the RNN evolution rule (Shi and Zhang, 2018) as below:


(32) $$\begin{array}{l} \dot{e}\left(t\right)=\frac{\text{d}e\left(t\right)}{\text{d}t}=-\zeta e\left(t\right), \end{array}$$

where the fixed convergence factor ζ > 0 has an important impact on the global exponential convergence rate. The larger the ζ chooses, the faster the convergence rate one acquires.

Then, the RNN evolution rule (32) can be further expanded as the following equation on account of the EE :


(33) $$\begin{array}{ll} \dot{H}\left(t\right)\chi \left(t\right) & +H\left(t\right)\dot{\chi }\left(t\right)+\dot{g}\left(t\right)=-\zeta \left[H\left(t\right)\chi \left(t\right)+g\left(t\right)\right]. \end{array}$$

For the handiness of figuring out the optimal solution to the TVQP problem (27)–(28), we reformulate (33) as


(34) $$\begin{array}{l} D\left(t\right)\dot{\chi }\left(t\right)=-V\left(t\right)\chi \left(t\right)-\varrho \left(t\right)-\zeta \left[H\left(t\right)\chi \left(t\right)+g\left(t\right)\right], \end{array}$$

where


$$\begin{array}{l} D\left(t\right)=\left[\begin{array}{ccc} U\left(t\right) & A^{\text{T}}\left(t\right) & B^{\text{T}}\left(t\right)\\ A\left(t\right) & 0_{12\times 12} & 0_{12\times 4n}\\ \left[\ell_{1}\left(t\right)-I_{4n\times 4n}\right]B\left(t\right) & 0_{4n\times 12} & I_{4n\times 4n}-\ell_{2}\left(t\right) \end{array}\right]\in {\mathbb{R}}^{\varpi \times \varpi }\\ V\left(t\right)=\left[\begin{array}{ccc} \dot{U}\left(t\right) & {\dot{A}}^{\text{T}}\left(t\right) & {\dot{B}}^{\text{T}}\left(t\right)\\ \dot{A}\left(t\right) & 0_{12\times 12} & 0_{12\times 4n}\\ \left[\ell_{1}\left(t\right)-I_{4n\times 4n}\right]\dot{B}\left(t\right) & 0_{4n\times 12} & 0_{4n\times 4n} \end{array}\right]\in {\mathbb{R}}^{\varpi \times \varpi }\\ \varrho \left(t\right)=\left[\begin{array}{c} \dot{\varphi }\left(t\right)\\ -\dot{c}\left(t\right)\\ \left[I_{4n\times 4n}-\ell_{1}\left(t\right)\right]\dot{d}\left(t\right) \end{array}\right]\in {\mathbb{R}}^{\varpi }, \end{array}$$

with


$$\begin{array}{l} \{\begin{array}{l} \ell_{1}\left(t\right)=\wedge \left[\Omega \left(t\right)˙v\left(t\right)\right]\\ \ell_{2}\left(t\right)=\wedge \left[\Omega \left(t\right)˙\mu \left(t\right)\right]\\ \Omega \left(t\right)={\left[v\left(t\right)˙v\left(t\right)+\mu \left(t\right)˙\mu \left(t\right)+\varepsilon_{3}\right]}^{-\frac{1}{2}},\varepsilon_{3}\to 0_{+}. \end{array} \end{array}$$

We treat (34) as a CRNN model. In order to facilitate its realization in computer system and digital hardware, the CTT-DRNN algorithm and the ATT-DRNN algorithm are introduced in the following subsections.

#### 2.3.1 CTT-DRNN algorithm

In this subsection, a conventional Taylor-type discretization formula is given, and the CTT-DRNN algorithm is obtained by combining it with the CRNN model (34).

Based on Hu et al. (2018), the three-step general Taylor-type discretization formula is formulated as follows:


(35) $$\begin{array}{l} {ẋ}_{k}=\frac{\left(-2a+1\right)x_{k+1}+6ax_{k}-\left(6a+1\right)x_{k-1}+2ax_{k-2}}{2\sigma }\\ +O\left(\sigma^{2}\right),\;k=2,3,4,..., \end{array}$$

where the argument *a* < 0; *k* is the updating index; σ > 0 is the fixed sampling period; *x*_*k*_ = *x*(*t*_*k*_) denotes the samping value of function *x*(*t*) at time instant *t*_*k*_ = *kσ*; *O*(σ^2^) is the TE.

By applying the three-step general Taylor-type discretization formula (35) to discretize the CRNN model (34), we can acquire CTT-DRNN algorithm as below:


(36) $$\begin{array}{ll} \chi_{k+1} & ≐\frac{6a\chi_{k}-\left(6a+1\right)\chi_{k-1}+2a\chi_{k-2}-2M_{k}\left[\sigma \left(-V_{k}\chi_{k}-\varrho_{k}\right)-h\left(H_{k}\chi_{k}+g_{k}\right)\right]}{2a-1} \end{array}$$

where symbol ≐ denotes the computational assignment operation; $M_{k},V_{k},H_{k},\varrho_{k},g_{k},$ and **χ**_*k*_ mean the instantaneous values of M(t),V(t),H(t),ϱ(t),g(t), and **χ**(*t*) sampling at time instant *t*_*k*_ with ***M***(*t*) denoting the pseudoinverse of ***D***(*t*); parameter *h* = σζ represents the solution step size generally set at the range of (0, 1).

#### 2.3.2 ATT-DRNN algorithm

According to the analysis of Subsection (2.3.1), on the one hand, the larger the fixed convergence factor ζ, the faster the global convergence rate of the system, thus we should naturally set ζ as large as possible at the beginning to ensure a sublime exponential converging capability of the CRNN model (34). On the other hand, it is recognized that the fixed argument σ as the sampling period is a significant factor affecting the convergence precision of the CTT-DRNN algorithm (36). Generally, the more remarkable convergence precision is guaranteed by a smaller value of σ taken at the initial stage. However, blindly setting a small value of σ may directly lead to an exiguous solution step size *h*, making it knotty for the solution process to converge rapidly or even proceed normally. Similarly, an excessively huge ζ also makes it hard to ensure a brilliant exactness of the algorithm due to incurring a gigantic solution step size *h*. It can be seen that the above situations are contradictory to each other. Moreover, according to the changes in system conditions, fixed parameters cannot meet the needs of different states. In view of this, to autonomously adjust the convergence factor ζ and the sampling period σ according to the actual convergence situation, and assure that the global state both has a remarkable convergence rate and outstanding convergence precision, a novel ATT-DRNN algorithm is designed as described in the following text.

First, according to the actual solution status, the adaptive sampling period σ_*k*_ = σ(*t*_*k*_) is designed as follows:


(37) $$\begin{array}{l} \sigma_{k}=\frac{q}{{\left(p+||e_{k}|{|}_{2}\right)}^{\delta }}, \end{array}$$

where fixed arguments *p, q* > 0 are applied to adjust the solution accuracy of the algorithm; variable argument δ is utilized to ensure the algorithm precision while adjusting the sampling period change rate; error $e_{k}=H_{k}\chi_{k}+g_{k}$ and symbol ||·||_2_ represents the 2-norm of a vector.

Accordingly, the adaptive convergence factor ζ_*k*_ = ζ(*t*_*k*_) is designed as follows:


(38) $$\begin{array}{l} \zeta_{k}=\frac{h{\left(p+||e_{k}|{|}_{2}\right)}^{\delta }}{q}, \end{array}$$

where variable argument δ is utilized to ensure the algorithm accuracy while adjusting the global convergence rate of the algorithm; *h* = σ_*k*_ζ_*k*_ is the same as that in (36).

Meanwhile, the corresponding continuous adaptive convergence factor ζ(*t*) can be written as follows:


(39) $$\begin{array}{l} \zeta \left(t\right)=\frac{h{\left(p+||e\left(t\right)|{|}_{2}\right)}^{\delta }}{q}. \end{array}$$

With the help of the continuous adaptive convergence factor ζ(*t*) (39), a novel RNN evolution rule can be written as follows:


(40) $$\begin{array}{l} \dot{e}\left(t\right)=\frac{\text{d}e\left(t\right)}{\text{d}t}=-\zeta \left(t\right)e\left(t\right). \end{array}$$

Thus, on the account of the EE (31), the novel RNN evolution rule (40) can be further expanded and reformulated as the ACRNN model:


(41) $$\begin{array}{l} \dot{\chi }\left(t\right)=M\left(t\right)\left\{-V\left(t\right)\chi \left(t\right)-\varrho \left(t\right)-\zeta \left(t\right)\left[H\left(t\right)\chi \left(t\right)+g\left(t\right)\right]\right\}, \end{array}$$

where the corresponding parameters are all the same as in the previous section.

Besides, by taking into account the adaptive sampling period σ_*k*_ (37), the adaptive three-step general Taylor-type discretization formula can be expressed as follows:


(42) $$\begin{array}{l} {ẋ}_{k}=\frac{\left(-2a+1\right)x_{k+1}+6ax_{k}-\left(6a+1\right)x_{k-1}+2ax_{k-2}}{2\sigma_{k}}\\ +O\left(\sigma_{k}^{2}\right),\;k=2,3,4,..., \end{array}$$

Then, we can acquire the ATT-DRNN algorithm by using the adaptive three-step general Taylor-type discretization formula (42) to discretize the ACRNN model (41), which can be written as follows:


(43) $$\begin{array}{l} \chi_{k+1}≐\frac{6a}{2a-1}\chi_{k}-\frac{6a+1}{2a-1}\chi_{k-1}+\frac{2a}{2a-1}\chi_{k-2}\\ -\frac{2}{2a-1}M_{k}\left[\sigma_{k}\left(-V_{k}\chi_{k}-\varrho_{k}\right)-h\left(H_{k}\chi_{k}+g_{k}\right)\right], \end{array}$$

where the solution step size *h* = σ_*k*_ζ_*k*_ is generally set at the range of (0, 1). Moreover, three initial state vectors **χ**_*k*_ with *k* = 0, 1, 2 are necessary to start up the proposed ATT-DRNN algorithm (43). The first one **χ**_0_ consists of **℧**_0_, **λ**_0_, and **μ**_0_, where **℧**_0_ is determined by the initial joint-velocity vectors of the LA and RA, while **λ**_0_ and **μ**_0_ are relatively arbitrarily set. The remaining initial state vectors can be generated by utilizing an adaptive Euler-type DRNN algorithm, which can be obtained by applying adaptive Euler forward formula to discretize the ACRNN model (41), i.e., $\chi_{k+1}≐\chi_{k}+\sigma_{k}{\dot{\chi }}_{k}$ with ${\dot{\chi }}_{k}=M_{k}\left[-V_{k}\chi_{k}-\varrho_{k}-\zeta_{k}\left(H_{k}\chi_{k}+g_{k}\right)\right]$.

*Remark 2.5:* By observing (37), it is evident that the adaptive sampling period σ_*k*_ continuously adjusts according to the changes in the RE ||***e***_*k*_||_2_, with an increase in the RE ||***e***_*k*_||_2_ and a decrease in the sampling period σ_*k*_, and vice versa.

*Remark 2.6:* By observing (38), it is evident that the adaptive convergence factor ζ_*k*_ continuously adjusts according to the changes in the RE ||***e***_*k*_||_2_, when the RE ||***e***_*k*_||_2_ increases, the adaptive convergence factor ζ_*k*_ grows, leading to a higher convergence rate, and vice versa.

*Remark 2.7:* The solution step size *h* procured through multiplying σ_*k*_ and ζ_*k*_ is always a positive constant. By observing Equations (37) and (38) simultaneously, it can be easily found that σ_*k*_ and ζ_*k*_ exhibit the reciprocal states to each other. That is to say, when the RE ||***e***_*k*_||_2_ is large, the algorithm will adjust and yield a smaller sampling period σ_*k*_ and a larger convergence factor ζ_*k*_ to guarantee a rapid convergence of the algorithm in an extremely short sampling time; on the contrary, when the RE ||***e***_*k*_||_2_ reduces, the algorithm will adaptively increase the sampling period σ_*k*_ and simultaneously decrease the convergence factor ζ_*k*_. By decreasing the sampling period and increasing the convergence rate, the algorithm can promptly complete the calculation and improve its computational efficiency. Therefore, the ATT-DRNN algorithm (43) can consider both computational accuracy and convergence efficiency during the calculation process.

## 3 Theoretical analyses and results

This section theoretically analyzes the convergence property of the ACRNN model (41) and the computational precision of the ATT-DRNN algorithm (43) for solving the TVQP problem (27)–(29).

*Theorem 1:* With the parameters *h, p, q*, δ > 0 of the continuous adaptive convergence factor ζ(*t*), the RE ||***e***(*t*)||_2_ generated by the ACRNN model (41) exponentially converges to zero in a large-scale manner with the exponential convergence rate at least being *hp*^δ^/*q*.

*Proof:* To begin with, by exploiting the EE (31), a Lyapunov function can be chosen as follows:


(44) $$\begin{array}{l} 𝕃\left(t\right)=\frac{||e\left(t\right)|{|}_{2}^{2}}{2}. \end{array}$$

Then, the time derivative of the function 𝕃(*t*) is obtained by referring to (40):


(45) $$\begin{array}{l} \dot{𝕃}\left(t\right)=e^{T}\left(t\right)\dot{e}\left(t\right)=-\zeta \left(t\right)||e\left(t\right)|{|}_{2}^{2}. \end{array}$$

Observing (44) and (45), one can draw the following conclusions.

(1) If and only if ***e***(*t*) = **0**, 𝕃(*t*) = 0; otherwise, 𝕃(*t*) > 0.

(2) If and only if ***e***(*t*) = **0**, $\dot{𝕃}\left(t\right)=0$; otherwise, $\dot{𝕃}\left(t\right)<0$.

In other words, the function *𝕃*(*t*) is positive definite and its derivative $\dot{𝕃}\left(t\right)$ is negative definite, which satisfies the Lyapunov stability theory conditions (Isidori, 1989). Thus, it can be concluded that the RE ||***e***(*t*)||_2_ converges to zero in a large-scale manner.

Second, by reconstructing and expanding (45), we acquire


(46) $$\begin{array}{l} \dot{𝕃}\left(t\right)=-2\frac{h{\left(p+||e\left(t\right)|{|}_{2}\right)}^{\delta }}{q}\frac{||e\left(t\right)|{|}_{2}^{2}}{2}=-2\frac{h{\left(p+||e\left(t\right)|{|}_{2}\right)}^{\delta }}{q}𝕃\left(t\right). \end{array}$$

Furthermore, based on (46), the following inequality can be further formulated as follows:


(47) $$\begin{array}{l} \dot{𝕃}\left(t\right)=-2\frac{h{\left(p+||e\left(t\right)|{|}_{2}\right)}^{\delta }}{q}𝕃\left(t\right)\leq -2\frac{hp^{\delta }}{q}𝕃\left(t\right). \end{array}$$

Attempting to figure out the inequality (47), we get


(48) $$\begin{array}{l} \frac{||e\left(t\right)|{|}_{2}^{2}}{2}\leq \frac{||e\left(0\right)|{|}_{2}^{2}}{2}\exp\left(-2\frac{hp^{\delta }}{q}t\right), \end{array}$$

and the inequality (48) can be further formulated as


(49) $$\begin{array}{l} ||e\left(t\right)|{|}_{2}\leq ||e\left(0\right)|{|}_{2}\exp\left(-\frac{hp^{\delta }}{q}t\right). \end{array}$$

Until now, in accordance with the inequality (49), we can conclude that the RE ||***e***(*t*)||_2_ of the ACRNN model (41) exponentially converges to zero in a large-scale manner with the exponential convergence rate at least being *hp*^δ^/*q*, which completes the proof.     ■

*Theorem 2:* With the parameters *h, p, q*, δ > 0 of the adaptive sampling period σ_*k*_, the ATT-DRNN algorithm (43) is zero-stable and convergent with the TE of order ***O***((*q*/*p*^δ^)^3^). In addition, the theoretical solution of TVES problem (30) converged by the ATT-DRNN algorithm (43) with a maximal steady-status RE $\lim_{k\to \infty }\text{sup}||e_{k+1}|{|}_{2}$ bing of order *O*((*q*/*p*^δ^)^3^).

*Proof:* First, drawing on the experience of Hu et al. (2018), it testified that the adaptive three-step general Taylor-type discretization formula (42) meets the conditions of zero-stable and convergent, which has a TE term ***O***(σ^2^).

By using the adaptive three-step general Taylor-type discretization formula (42) to discretize the ACRNN model (41), the new ATT-DRNN algorithm (43) can be rewritten as follows:


(50) $$\begin{array}{l} \chi_{k+1}=\frac{6a}{2a-1}\chi_{k}-\frac{6a+1}{2a-1}\chi_{k-1}+\frac{2a}{2a-1}\chi_{k-2}\\ -\frac{2}{2a-1}M_{k}\left[\sigma_{k}\left(-V_{k}\chi_{k}-\varrho_{k}\right)-h\left(H_{k}\chi_{k}+g_{k}\right)\right]+O\left(\sigma_{k}^{3}\right),\; \end{array}$$

where $O\left(\sigma_{k}^{3}\right)$ is the TE term.

Due to the development process recommended above, it is distinct that the ATT-DRNN algorithm (43) originating from (50) is also zero-stable and similarly convergent with the TE of order $O\left(\sigma_{k}^{3}\right)$. Therefore, we get


(51) $$\begin{array}{l} \underset{k\to \infty }{\lim}\sigma_{k}=\underset{k\to \infty }{\lim}\frac{q}{{\left(p+||e_{k}|{|}_{2}\right)}^{\delta }}=\frac{q}{p^{\delta }}, \end{array}$$

which means that the TE term for the ATT-DRNN algorithm (43) is ***O***((*q*/*p*^δ^)^3^).

Then, based on the ATT-DRNN algorithm (43), the theoretical solution $\chi_{k+1}^{*}$ of TVES problem (30) can be expressed as follows:


(52) $$\begin{array}{l} \chi_{k+1}^{*}=\chi_{k+1}+O\left({\left(q/p^{\delta }\right)}^{3}\right). \end{array}$$

In addition, it is known that the theoretical solution $\chi_{k+1}^{*}$ of the TVES problem (30) satisfies $H_{k+1}\chi_{k+1}^{*}+g_{k+1}=0$. Thus, by combining (51) with (52), we can draw the following conclusion:


(53) $$\begin{array}{l} \;\underset{k\to \infty }{\lim}\text{sup}||e_{k+1}|{|}_{2}\\ =\underset{k\to \infty }{\lim}\text{sup}||H_{k+1}\chi_{k+1}+g_{k+1}|{|}_{2}\\ =\underset{k\to \infty }{\lim}\text{sup}||H_{k+1}\chi_{k+1}^{*}+g_{k+1}-H_{k+1}O\left(\sigma_{k}^{3}\right)|{|}_{2}\\ =\underset{k\to \infty }{\lim}\text{sup}||H_{k+1}O\left(\sigma_{k}^{3}\right)|{|}_{2}\\ \leq \underset{k\to \infty }{\lim}\text{sup}\left(||H_{k+1}|{|}_{\text{F}}||O\left(\sigma_{k}^{3}\right)|{|}_{2}\right)=O\left({\left(q/p^{\delta }\right)}^{3}\right). \end{array}$$

Based on (53), it can be concluded that the maximal steady-status RE $\lim_{k\to \infty }\text{sup}||e_{k+1}|{|}_{2}$ generated by the ATT-DRNN algorithm (43) is *O*((*q*/*p*^δ^)^3^). Thus, we complete the proof.     ■

## 4 Illustrative examples

In this section, a numerical simulation example is provided first and explored to state explicitly the remarkable competence of the devised ATT-DRNN algorithm (43) when tackling the TVQP problem (27)–(29). Then, two examples of dual-arm robot control are provided to demonstrate the effectiveness of the devised ATT-DRNN algorithm (43) in addressing the proposed DAPCMC scheme (27)–(29). Meanwhile, we utilize the CTT-DRNN algorithm (36) and the CET-DRNN algorithm in Wu and Zhang (2023) for comparisons to show the superior performance of the devised ATT-DRNN algorithm (43). To help readers understand the algorithm implementation process, the pseudo-code of the proposed ATT-DRNN algorithm (43) for addressing the DAPCMC scheme (27)–(29) is presented in Algorithm 1.

**Algorithm 1:** Pseudo-code of the proposed ATT-DRNN algorithm (43) for addressing the DAPCMC scheme (27)–(29).

1. **Set**: α, β, ξ, *a*, *h*, *p*, *q*, δ, *T*_e_, γ, $z_{L}^{\pm }$, $z_{R}^{\pm }$, ${\dot{z}}_{L}^{\pm }$, ${\dot{z}}_{R}^{\pm }$;
2. **Initialize**: $z_{L}\left(0\right)$, $z_{R}\left(0\right)$, ${℧}_{L}\left(0\right)$, ${℧}_{R}\left(0\right)$, **λ**(0), **μ**(0), *t*(0);
3. **while** *t*_*k*_ ≤ *T*_e_ **do**
4. Generate ${℧}_{L}^{\pm }\left(t_{k}\right)$, ${℧}_{R}^{\pm }\left(t_{k}\right)$ from the JCLS;
5. Compute $H\left(t_{k}\right)$, **χ**(*t*_*k*_), $g\left(t_{k}\right)$, ***M***(*t*_*k*_), ***V***(*t*_*k*_), ***ϱ**(t_k_), **V**(t_k_), σ(t_k_), ζ(t_k_);*
6. **if** *k* = 0, 1 **then**
7. Compute **χ**(*t*_*k*+1_) via the adaptive Euler-type DRNN algorithm: $\;\chi \left(t_{k+1}\right)≐\chi \left(t_{k}\right)+\sigma \left(t_{k}\right)\dot{\chi }\left(t_{k}\right);$
8. **else**
9. Compute **χ**(*t*_*k*+1_) via the ATT-DRNN algorithm: $\begin{array}{c} \;\chi \left(t_{k+1}\right)≐\frac{6a}{2a-1}\chi \left(t_{k}\right)-\frac{6a+1}{2a-1}\chi \left(t_{k-1}\right)+\frac{2a}{2a-1}\chi \left(t_{k-2}\right)\\ -\frac{2}{2a-1}\sigma \left(t_{k}\right)\dot{\chi }\left(t_{k}\right);\; \end{array}$ 10. Obtain ${℧}_{L}\left(t_{k+1}\right)$ from the first *n* elements of **χ**(*t*_*k*+1_) and ${℧}_{R}\left(t_{k+1}\right)$ from the *n* + 1
to 2*n* elements of **χ**(*t*_*k*+1_);
11. **if** *k* = 0 **then**
12. Compute $z_{L}\left(t_{k+1}\right)=z_{L}\left(t_{k}\right)+\sigma \left(t_{k}\right){℧}_{L}\left(t_{k+1}\right)$,
$z_{R}\left(t_{k+1}\right)=z_{R}\left(t_{k}\right)+\sigma \left(t_{k}\right){℧}_{R}\left(t_{k+1}\right)$;
13. **else**
14. Compute $z_{L}\left(t_{k+1}\right)=\frac{4}{3}z_{L}\left(t_{k}\right)-\frac{1}{3}z_{L}\left(t_{k-1}\right)+\frac{2}{3}\sigma \left(t_{k}\right){℧}_{L}\left(t_{k+1}\right)$,
$z_{R}\left(t_{k+1}\right)=\frac{4}{3}z_{R}\left(t_{k}\right)-\frac{1}{3}z_{R}\left(t_{k-1}\right)+\frac{2}{3}\sigma \left(t_{k}\right){℧}_{R}\left(t_{k+1}\right)$;
15. s Update *t*_*k*+1_ = *t*_*k*_ + σ(*t*_*k*_);
16. **end**

### 4.1 Numerical simulation verification

A specific TVQP problem with equality and inequality constraints is provided, the details of which are outlined below:


(54) $$\begin{array}{l} {\text{min}.}_{℧\left(t\right)}\left[\cos\left(3t\right)/6+1\right]{℧}_{1}^{2}\left(t\right)+\sin\left(t\right){℧}_{1}\left(t\right){℧}_{2}\left(t\right)\\ +\cos\left(3t\right){℧}_{1}\left(t\right){℧}_{3}\left(t\right)+2\sin\left(t\right){℧}_{1}\left(t\right)\\ +\left[\sin\left(3t\right)/6+1\right]{℧}_{2}^{2}\left(t\right)+\left[\sin\left(2t\right)+1\right]{℧}_{2}\left(t\right){℧}_{4}\left(t\right)\\ +2\cos\left(t\right){℧}_{2}\left(t\right)-\left[\cos\left(t\right)/2+1/2\right]{℧}_{3}^{2}\left(t\right)\\ +\left[\cos\left(2t\right){℧}_{3}\left(t\right){℧}_{4}\left(t\right)\right]/2+\sin\left(2t\right){℧}_{3}\left(t\right)\\ -\left[\sin\left(t\right)/2+1/2\right]{℧}_{4}^{2}\left(t\right)+\cos\left(2t\right){℧}_{4}\left(t\right)\\ \;\\ \text{s.t.}-t\sin\left(t/2\right){℧}_{1}\left(t\right)+\left\{\left[4\cos\left(t/2\right)\right]/5+1/5\right\}{℧}_{2}\left(t\right)=t\sin\left(2t+1\right)\\ -\left[3\cos\left(9t/10\right)\right]/2{℧}_{3}\left(t\right)+\sin\left(9t/10\right){℧}_{4}\left(t\right)=-\cos\left(3t/2\right)/2-3/10\\ -0.2\sin\left(3t\right)-1.2\leq {℧}_{1}\left(t\right),{℧}_{2}\left(t\right),{℧}_{3}\left(t\right),{℧}_{4}\left(t\right)\leq 0.2\sin\left(3t\right)+1.2 \end{array}$$

where $℧\left(t\right)={\left[{℧}_{1}\left(t\right),{℧}_{2}\left(t\right),{℧}_{3}\left(t\right),{℧}_{4}\left(t\right)\right]}^{T}$. By referring to the standard form of the TVQP problem (27)–(29), the corresponding coefficients are as follows:


$$\begin{array}{l} U\left(t\right)=\left[\begin{array}{cccc} \cos\left(3t\right)/3+2 & \sin\left(t\right) & \cos\left(3t\right) & 0\\ \sin\left(t\right) & \sin\left(3t\right)/3+2 & 0 & \sin\left(2t\right)+1\\ \cos\left(3t\right) & 0 & -\cos\left(t\right)-1 & \cos\left(2t\right)/2\\ 0 & \sin\left(2t\right)+1 & \cos\left(2t\right)/2 & -\sin\left(t\right)-1 \end{array}\right]\in {\mathbb{R}}^{4\times 4}\; \end{array}$$

$$\begin{array}{l} \varphi \left(t\right)=\left[\begin{array}{cccc} \sin\left(t\right) & \cos\left(t\right) & \sin\left(2t\right) & \cos\left(2t\right) \end{array}\right]\in {\mathbb{R}}^{4} \end{array}$$

$$\begin{array}{l} A\left(t\right)=\left[\begin{array}{cccc} -t\sin\left(t/2\right) & \left[4\cos\left(t/2\right)\right]/5+1/5 & 0 & 0\\ 0 & 0 & -\left[3\cos\left(9t/10\right)\right]/2 & \sin\left(9t/10\right)\\ \; \end{array}\right]\in {\mathbb{R}}^{2\times 4} \end{array}$$

$$\begin{array}{l} B\left(t\right)=\left[\begin{array}{c} \;I_{4\times 4}\\ -I_{4\times 4} \end{array}\right]\in {\mathbb{R}}^{8\times 4}\\ c\left(t\right)=\left[\begin{array}{c} t\sin\left(2t+1\right)\\ -\cos\left(3t/2\right)/2-3/10 \end{array}\right]\in {\mathbb{R}}^{2}\\ d\left(t\right)={\left[\begin{array}{c} 0.2\sin\left(3t\right)+1.2 \end{array}\right]}_{8\times 1}\in {\mathbb{R}}^{8}. \end{array}$$

To successfully address the above TVQP problem (54) using the ATT-DRNN algorithm (42), we set parameters *h* = 0.1, *p* = 5, *q* = 0.05, and γ = 0.001; the initial values of **℧**(0), **λ**(0), and **μ**(0) are set to random values at the range of (0, 0.001). Then, the entire simulation calculation time in the program is uniformly set to *T*_e_ = 4 s. Besides, we set the fixed sampling period σ = 0.01 s for the CTT-DRNN algorithm (36) and the CET-DRNN algorithm in Wu and Zhang (2023).

**Figure 2:**
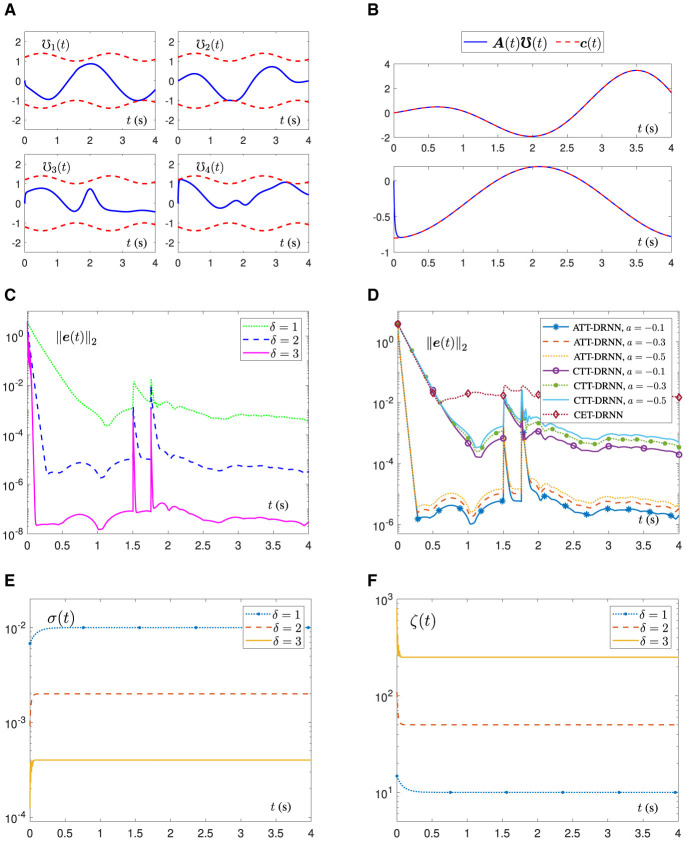
Numerical simulation results of the ATT-DRNN (43), CTT-DRNN (36), and CET-DRNN in Wu and Zhang (2023) algorithms for addressing the TVQP problem (53), separately. **(A)** Status vector **℧**(*t*) under inequality constraint generated by the ATT-DRNN algorithm (43) with *a* = −0.3 and δ = 2. **(B)** ***A***(*t*)**℧**(*t*) under equality constraint *c*(*t*) obtained by the ATT-DRNN algorithm (43) with *a* = −0.3 and δ = 2. **(C)** RE ||***e***(*t*)||_2_ generated by the ATT-DRNN algorithm (43) with *a* = −0.3 and different δ. **(D)** RE ||***e***(*t*)||_2_ by three algorithms with different *a* and δ = 2. **(E)** Adaptive sampling period σ(*t*) with different δ. **(F)** Adaptive convergence factor ζ(*t*) with different δ.

Figure 2A shows the element trajectories of the status vector **℧**(*t*) generated by the ATT-DRNN algorithm (43) with *a* = −0.3 and δ = 2, which are strictly confined to the ranges of inequality constraints. Meanwhile, it can be seen from Figure 2B that the equality constraint ***A***(*t*)**℧**(*t*) of the TVQP problem (54) can be promptly satisfied and can consistently maintain this state. To save space, the solving states of the proposed algorithm (43) with different *a* and δ, as well as similar figures of other algorithms, are omitted.

In order to research the impact of δ on the solving results of the ATT-DRNN algorithm (43), the variation trajectory of the RE ||***e***(*t*)||_2_ when taking different δ with *a* = −0.3 is exhibited in Figure 2C. As we can see that, when entering the steady state, the RE ||***e***(*t*)||_2_ maintains at around 10^−4^ with δ = 1, 10^−6^ with δ = 2, and 10^−8^ with δ = 3. In other words, as the setting value of δ increases, the convergence speed of the ATT-DRNN algorithm (43) is accelerated, and the solution precision is higher.

To demonstrate the excellent performance of the proposed ATT-DRNN algorithm (43) compared with other conventional algorithms, we further investigate the REs ||***e***(*t*)||_2_ generated by the algorithms of ATT-DRNN (43) with δ = 2, CTT-DRNN (36) and CET-DRNN in Wu and Zhang (2023) by figuring out the TVQP problem (54), respectively. The REs ||***e***(*t*)||_2_ synthesized by these three algorithms with different *a* are displayed in Figure 2D. It can be seen that the RE ||***e***(*t*)||_2_ generated by the ATT-DRNN algorithm (43) with δ = 2 and different *a* values can converge as small as 10^−6^ in approximately 0.3 s. The REs ||***e***(*t*)||_2_ generated by the CTT-DRNN algorithm (36) with different *a* values can converge to roughly 10^−4^ in 1 s. The RE ||***e***(*t*)||_2_ generated by the CET-DRNN algorithm in Wu and Zhang (2023) merely converges to around 10^−2^ in 0.5 s. Overall, the solution accuracy and convergence rate of the ATT-DRNN algorithm (43) are superior to the other two conventional algorithms. Besides, it can be concluded that the computing precision of the ATT-DRNN algorithm (43) is higher with the smaller absolute value of *a* chosen. In addition, the variation curves of the adaptive sampling period σ(*t*) and the adaptive convergence factor ζ(*t*) with different δ values are portrayed in Figures 2E, F, separately, which indicate that σ(*t*) and ζ(*t*) can converge and stabilize to their corresponding values in an extremely short time. The greater the δ chosen, the smaller the final stable value of σ(*t*) and the larger the final stable value of ζ(*t*). Furthermore, during the solving process, as the RE ||***e***(*t*)||_2_ rapidly converges and decreases at the beginning stage, the change of σ(*t*) is inversely proportional to it, and the change of ζ(*t*) is directly proportional to it, which is consistent with our previous analysis conclusions from *Remark 2.5* to *Remark 2.7*.

In summary, the several situations above confirm that the ATT-DRNN algorithm (43) has an excellent ability to solve the TVQP problem. Compared with other conventional algorithms, the proposed algorithm has faster convergence speed and higher precision.

### 4.2 Control case I of dual-arm robot

For this fraction, we establish a DAPCMC scheme consisting of two UR5 robotic arms placed on the contralateral side, controlled by the ATT-DRNN algorithm (43) for dual heart-shaped trajectory tracking. In addition, the UR5 robotic arm is a sensitive lightweight 6-DOF robot, which has a small footprint and can be directly installed in a narrow workspace to complete tasks with high sensitivity requirements (Vivas and Sabater, 2021).

According to the design form of the DAPCMC scheme (27), (28), we establish a particular TVQP problem for a UR5 dual-arm robot consisting of two 6-DOF (with *n* = 6) UR5 robotic arms. In this scheme, the LA and RA's initial joint-angle vectors are set as $z_{L}\left(0\right)={\left[0,\pi /12,-4\pi /9,13\pi /36,\pi /2,0\right]}^{\text{T}}$ rad and $z_{R}\left(0\right)={\left[0,-\pi /12,4\pi /9,-13\pi /36,-\pi /2,0\right]}^{\text{T}}$ rad, respectively; the LA and RA's initial joint-velocity vectors are set as ${\dot{z}}_{L}\left(0\right)={\dot{z}}_{R}\left(0\right)={\left[0\right]}_{6\times 1}$ rad/s. We set the design parameters as *h* = 0.2, *p* = 10, *q* = 0.2, and δ = 2. Then, the other correlative parameters are taken as *a* = −0.1, ξ = 5, and γ = 0.001; λ and μ are set to random values at the range of (0, 0.001); α and β for two robotic arms are uniformly set as 0.8 and 0.1. Besides, we set the fixed sampling period σ = 0.01 s for the CTT-DRNN algorithm (36) and the CET-DRNN algorithm in Wu and Zhang (2023). Moreover, the D-H parameters of the UR5 robotic arm and its joint-angle and joint-velocity physical limits in the DAPCMC scheme (27)–(29) are exhibited in Table 2.

**Table 2:** The D-H parameters of UR5 robotic arm and its joint-angle and joint-velocity physical limits in the DAPCMC scheme (27)–(29).

**Joint**	** $\tilde{\alpha }\;\left(\text{rad}\right)$ **	** $\tilde{a}\;\left(\text{m}\right)$ **	** $\tilde{d}\;\left(\text{m}\right)$ **	** z (rad) **	** $z^{+}\;\left(\text{rad}\right)$ **	** $z^{-}\;\left(\text{rad}\right)$ **	** ${\dot{z}}^{+}\;\left(\text{rad/s}\right)$ **	** ${\dot{z}}^{-}\;\left(\text{rad/s}\right)$ **
1	1.5708	0	0.0892	$z_{1}$	1.5708	−1.5708	0.285	−0.285
2	0	−0.4250	0	$z_{2}$	0	−3.1416	0.285	−0.285
3	0	−0.3923	0	$z_{3}$	0	−3.1416	0.285	−0.285
4	1.5708	0	0.1092	$z_{4}$	1.5708	−1.5708	0.285	−0.285
5	−1.5708	0	0.0948	$z_{5}$	3.1416	0	0.285	−0.285
6	0	0	0.0825	$z_{6}$	1.5708	−1.5708	0.285	−0.285

**Figure 3:**
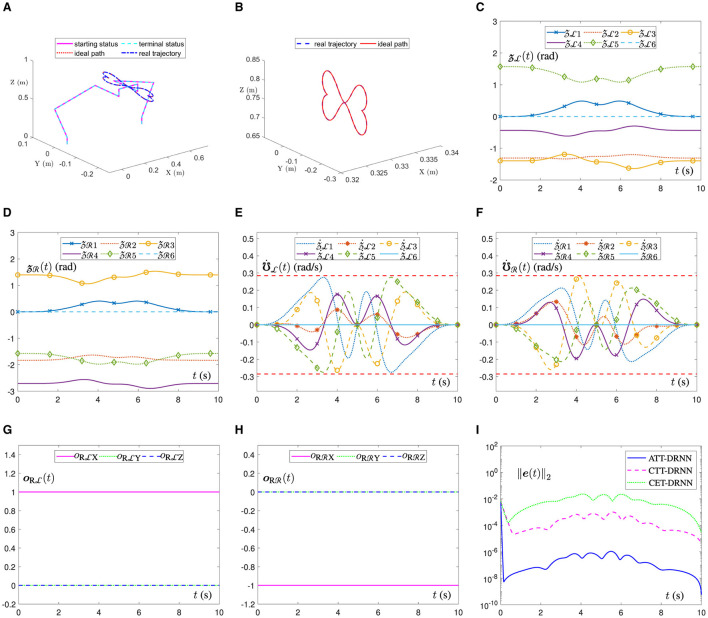
The ATT-DRNN algorithm (43) controls the posture collaboration motion of the UR5 dual-arm robot with two arms placed on the contralateral side for the dual heart-shaped trajectory tracking. **(A)** Starting and terminal statuses of the dual-arm robot and the end-executor real and ideal trajectories in 3D space. **(B)** Outlines of real trajectory and ideal path in a 3D space. **(C)** Variations of the LA joint angles $z_{L}\left(t\right)$. **(D)** Variations of the RA joint angles $z_{R}\left(t\right)$. **(E)** Variations of the LA joint velocities ${\dot{℧}}_{L}\left(t\right)$. **(F)** Variations of the RA joint velocities ${\dot{℧}}_{R}\left(t\right)$. **(G)** Variations of the LA end-executor orientation $o_{\text{R}L}\left(t\right)$. **(H)** Variations of the RA end-executor orientation $o_{\text{R}R}\left(t\right)$. **(I)** The RE ||***e***(*t*)||_2_ generated by three different algorithms.

Figure 3A illustrates the movement trajectory outlines of the dual-arm robot in a 3D space. It can be observed that the end-executor real trajectory is unanimous with the ideal path and that the joint-angle terminal status also perfectly overlaps with the starting status for each side of the dual robotic arms, which can be further confirmed by Figures 3C, D. Similarly, in Figure 3B, the ATT-DRNN algorithm (43) controls the dual-arm robot to achieve the posture collaboration motion and accomplish the dual heart-shaped path tracking. Finally, Figures 3E, F outline the joint-velocity variation curves for the joints of the left and right robotic arms. Clearly, all the joint-velocity values are not beyond the joint-velocity physical limits set at the beginning. Besides, the end-executor orientation variation curves are shown in Figures 3G, H, which remain constant during the task execution. Furthermore, in Figure 3I, the RE ||***e***(*t*)||_2_ generated by the ATT-DRNN algorithm (43) for addressing the DAPCMC scheme (27)-(29) maintains at around 10^−7^. By contrast, the RE ||***e***(*t*)||_2_ generated by the CTT-DRNN algorithm (36) keeps at roughly 10^−4^, and that generated by the CET-DRNN algorithm in Wu and Zhang (2023) can merely remain at about 10^−2^.

**Figure 4:**
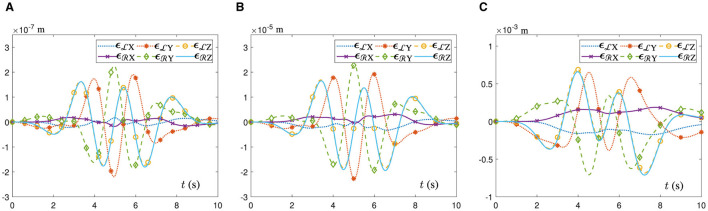
Variations of the end-executor position error when the UR5 dual-arm robot with two arms placed on the contralateral side to achieve the posture collaboration motion for the dual heart-shaped trajectory tracking. **(A)** Generated by the ATT-DRNN algorithm (43). **(B)** Generated by the CTT-DRNN algorithm (36). **(C)** Generated by the CET-DRNN algorithm in Wu and Zhang (2023).

In addition, Figure 4 shows the position error variation curves of the end-executor when the UR5 dual-arm robot tracks the dual heart-shaped trajectory under the control of different algorithms. In Figure 4A, the dual-arm robot controlled by the ATT-DRNN algorithm (43) can accomplish the trajectory following task accurately with the maximal position error of the end-executor being less than 2.3 × 10^−7^ m. In Figure 4B, the dual-arm robot controlled by the CTT-DRNN algorithm (36) can realize the maximal tracking error of the end-executor no more than 2.3 × 10^−5^ m. In Figure 4C, the dual-arm robot controlled by the CET-DRNN algorithm in Wu and Zhang (2023) can merely ensure that the position error of the end-executor is within 7 × 10^−4^ m.

**Figure 5:**
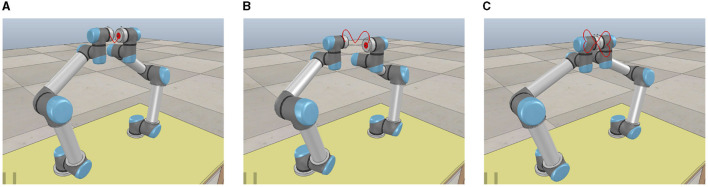
Snapshots of the UR5 dual-arm robot with two arms placed on the contralateral side during the trajectory tracking task of the dual heart-shaped path via the ATT-DRNN algorithm (43) solving the DAPCMC scheme (27)–(29). **(A)** Capturing at the starting moment. **(B)** Capturing at the intermediate moment. **(C)** Capturing at the terminal moment.

To further simulate the movement status of the dual-arm robot vividly and intuitively in the physical scene, we utilize a virtual robot experiment platform (i.e., CoppeliaSim 2020) to show the real-time status of the dual-arm robot following the ideal paths with the help of the ATT-DRNN algorithm (43) in solving the DAPCMC scheme (27)–(29). Snapshots describing the movement process (i.e., starting moment, intermediate moment, and terminal moment) are shown in Figure 5.

### 4.3 Control case II of dual–arm robot

For this fraction, we establish a DAPCMC scheme consisting of two UR5 robotic arms placed on the identical side, controlled by the ATT-DRNN algorithm (43) for the heart-shaped and auspicious cloud trajectory tracking.

According to the design form of the DAPCMC scheme (27), (28), we establish another particular TVQP problem for a UR5 dual-arm robot consisting of two 6-DOF UR5 robotic arms. In this scheme, the LA and RA's initial joint-angle vectors are set as $z_{L}\left(0\right)=z_{R}\left(0\right)={\left[0,-\pi /12,-2\pi /3,\pi /4,-\pi /2,0\right]}^{\text{T}}$ rad; the LA and RA's initial joint-velocity vectors are set as ${\dot{z}}_{L}\left(0\right)={\dot{z}}_{R}\left(0\right)={\left[0\right]}_{6\times 1}$ rad/s. We set the design parameters as *h* = 0.2, *p* = 5, *q* = 0.05, and δ = 2. Then, the other correlative parameters are taken as *a* = −0.5, ξ = 5, and γ = 0.001; λ and μ are set to random values at the range of (0, 0.001); α and β for two robotic arms are uniformly set as 0.8 and 0.1. Additionally, the joint-angle and joint-velocity physical limits in the DAPCMC scheme (27)-(28) are separately set as follows: $z_{L}^{+}=0.3$ rad/s, $z_{L}^{-}=-0.3$ rad/s, $z_{R}^{-}=0.54$ rad/s, and $z_{R}^{-}=-0.54$ rad/s.

**Figure 6:**
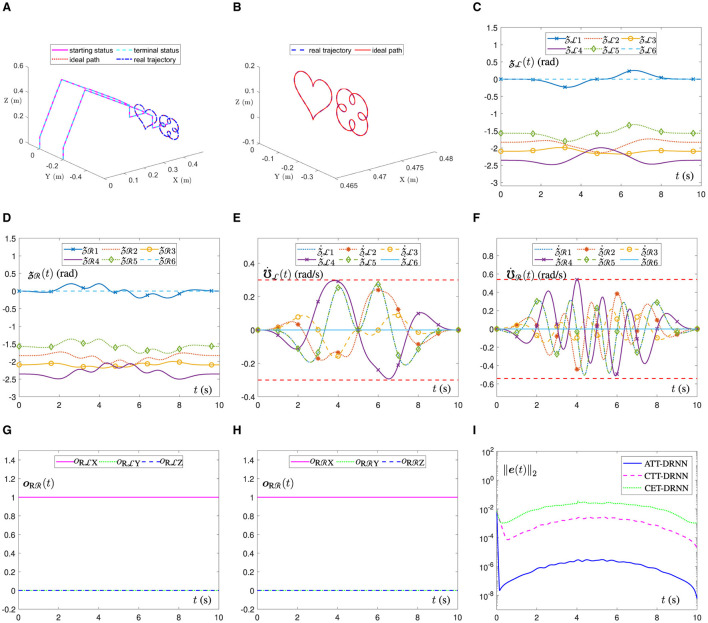
The ATT-DRNN algorithm (43) controls the posture collaboration motion of the UR5 dual-arm robot with two arms placed on the identical side for the heart-shaped and auspicious cloud trajectory tracking. **(A)** Starting and terminal statuses of the dual-arm robot and the end-executor real and ideal trajectories in a 3D space. **(B)** Outlines of real trajectory and ideal path in a 3D space. **(C)** Variations of the LA joint angles $z_{L}\left(t\right)$. **(D)** Variations of the RA joint angles $z_{R}\left(t\right)$. **(E)** Variations of the LA joint velocities ${\dot{℧}}_{L}\left(t\right)$. **(F)** Variations of the RA joint velocities ${\dot{℧}}_{R}\left(t\right)$. **(G)** Variations of the LA end-executor orientation $o_{\text{R}L}\left(t\right)$. **(H)** Variations of the RA end-executor orientation $o_{\text{R}R}\left(t\right)$. **(I)** The RE ||***e***(*t*)||_2_ generated by three different algorithms.

Figure 6A shows the movement trajectory outlines of the dual-arm robot in a 3D space. It is evident that the end-executor real trajectory aligns seamlessly with the ideal path. Moreover, the terminal statuses of the joint angles for both robotic arms precisely coincide with their initial ones, as corroborated by the results presented in Figures 6C, D. Similarly, in Figure 6B, the ATT-DRNN algorithm (43) controls the dual-arm robot to realize the posture collaboration motion and accomplish the task of tracking the heart-shaped and auspicious cloud trajectories separately. Subsequently, Figures 6E, F delineate the joint-velocity profiles of the left and right robotic arms. It is apparent that none of the joint-velocity values exceed the predetermined physical limits initially determined. Besides, the end-executor orientation variation curves are shown in Figures 6G, H, which maintain a constant state during the task execution. Furthermore, in Figure 6I, the RE ||***e***(*t*)||_2_ generated by the ATT-DRNN algorithm (43) maintains at approximately 10^−6^ and converges to a extremely small value of 10^−8^. By contrast, the RE ||***e***(*t*)||_2_ generated by the CTT-DRNN algorithm (36) keeps at roughly 10^−3^ and converges to about 10^−5^ and that generated by the CET-DRNN algorithm in Wu and Zhang (2023) can merely converge to about 10^−3^.

**Figure 7:**
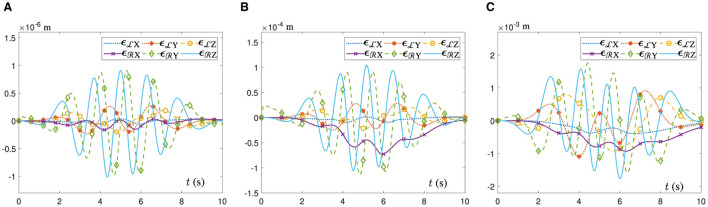
Variations of the end-executor position error when the UR5 dual-arm robot with two arms placed on the identical side to achieve the posture collaboration motion for the heart-shaped and auspicious cloud trajectory tracking. **(A)** Generated by the ATT-DRNN algorithm (43). **(B)** Generated by the CTT-DRNN algorithm (36). **(C)** Generated by the CET-DRNN algorithm (Wu and Zhang, 2023).

In addition, Figure 7 shows the position error variation curves of the end-executor when the UR5 dual-arm robot tracks the heart-shaped and auspicious cloud trajectory under the control of different algorithms. In Figure 7A, the dual-arm robot controlled by the ATT-DRNN algorithm (43) can accomplish the trajectory following task accurately with the maximal position error of the end-executor being less than 1.0 × 10^−6^ m. In Figure 7B, the dual-arm robot controlled by the CTT-DRNN algorithm (36) can realize the maximal position error of the end-executor no more than 1.2 × 10^−4^ m. In Figure 7C, the dual-arm robot controlled by the CET-DRNN algorithm in Wu and Zhang (2023) can merely ensure that the position error of the end-executor is within 1.8 × 10^−3^ m.

**Figure 8:**
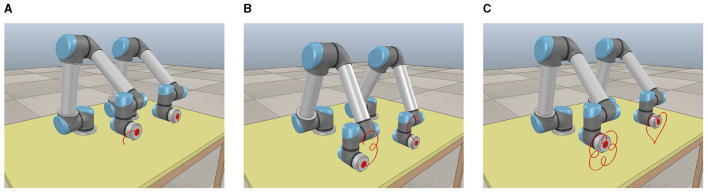
Snapshots of the UR5 dual-arm robot with two arms placed on the identical side during the trajectory tracking task of the heart-shaped and auspicious cloud path via the ATT-DRNN algorithm (43) solving the DAPCMC scheme (27)-(29). **(A)** Capturing at the starting moment. **(B)** Capturing at the intermediate moment. **(C)** Capturing at the terminal moment.

To further simulate the movement status of the dual-arm robot vividly and intuitively in the physical scene, we utilize the virtual robot experiment platform to show the real-time status of the dual-arm robot following the ideal paths with the help of the ATT-DRNN algorithm (43) in solving the DAPCMC scheme (27)–(29). Snapshots describing the movement process (i.e., starting moment, intermediate moment, and terminal moment) are shown in Figure 8.

In summary, the aforementioned two control cases of dual-arm robots substantiate that the proposed DAPCMC scheme (27)–(29) and its corresponding ATT-DRNN algorithm (43) can be utilized for the posture collaboration control of the industrial robots with joint physical limits considered and further demonstrate the potential of the proposed scheme and algorithm to optimize the efficiency and precision of repetitive trajectory tracking in practical applications.

## 5 Conclusion

In this study, the ATT-DRNN algorithm (43) has been devised for solving the DAPCMC scheme (27)–(29) with a novel JLCS (7), (8). Additionally, theoretical analyses and results have indicated the excellent performance of the ATT-DRNN algorithm (43) and the ACRNN model (41) in terms of the convergence rate and precision. Then, three illustrative examples with comparisons have further demonstrated that the proposed DAPCMC scheme (27)–(29) in a 3D space with the innovative JLCS (7), (8) offers a new solution measure for realizing the posture collaboration motion control of constrained dual-arm robots and accomplishing repetitive trajectory following missions, and it can be worked out by the ATT-DRNN algorithm (43) efficiently and accurately.

Finally, some possible research directions in the future are put forward.

- The whole design process of the ATT-DRNN algorithm (43) is set in an ideal noiseless environment. Therefore, enhancing the ATT-DRNN algorithm (43) with relevant anti-noise technologies to make it possess strong robustness in various noise environments is an interesting future research direction.
- The ATT-DRNN algorithm (43) involves an explicit inverse operation, which is computationally expensive. Thus, proposing an inverse-free ATT-DRNN algorithm is another future research direction.
- Two robotic arms of the same model are used to form a dual-arm robot in this study. Thus, achieving the collaboration motion control by composing a heterogenous multi-arm robot system is a meaningful future research direction.
- Popularizing the ATT-DRNN design scheme to more kinds of engineering applications (e.g., UAV flight control) is also a significant future research direction.

## Data availability statement

The original contributions presented in the study are included in the article/supplementary material, further inquiries can be directed to the corresponding author.

## Author contributions

YZ: Data curation, Formal analysis, Software, Validation, Visualization, Writing – original draft. YH: Funding acquisition, Project administration, Resources, Supervision, Writing – review & editing. BQ: Conceptualization, Funding acquisition, Investigation, Methodology, Resources, Supervision, Writing – review & editing.
